# Neural Correlates of Vestibular Processing During a Spaceflight Analog With Elevated Carbon Dioxide (CO_2_): A Pilot Study

**DOI:** 10.3389/fnsys.2019.00080

**Published:** 2020-01-10

**Authors:** Kathleen E. Hupfeld, Jessica K. Lee, Nichole E. Gadd, Igor S. Kofman, Yiri E. De Dios, Jacob J. Bloomberg, Ajitkumar P. Mulavara, Rachael D. Seidler

**Affiliations:** ^1^Department of Applied Physiology and Kinesiology, University of Florida, Gainesville, FL, United States; ^2^German Aerospace Center, Institute of Aerospace Medicine, Cologne, Germany; ^3^KBR, Houston, TX, United States; ^4^NASA Johnson Space Center, Houston, TX, United States; ^5^Department of Neurology, University of Florida, Gainesville, FL, United States

**Keywords:** vestibular, fMRI, head-down-tilt bed rest (HDBR), carbon dioxide (CO_2_), spaceflight

## Abstract

Astronauts return to Earth from spaceflight missions with impaired mobility and balance; recovery can last weeks postflight. This is due in large part to the altered vestibular signaling and sensory reweighting that occurs in microgravity. The neural mechanisms of spaceflight-induced vestibular changes are not well understood. Head-down-tilt bed rest (HDBR) is a common spaceflight analog environment that allows for study of body unloading, fluid shifts, and other consequences of spaceflight. Subjects in this context still show vestibular changes despite being in Earth’s gravitational environment, potentially due to sensory reweighting. Previously, we found evidence of sensory reweighting and reduced neural efficiency for vestibular processing in subjects who underwent a 70-day HDBR intervention. Here we extend this work by evaluating the impact of HDBR paired with elevated carbon dioxide (CO_2_) to mimic International Space Station conditions on vestibular neural processing. Eleven participants (6 males, 34 ± 8 years) completed 30 days of HDBR combined with 0.5% atmospheric CO_2_ (HDBR + CO_2_). Participants underwent six functional magnetic resonance imaging (fMRI) sessions pre-, during, and post- HDBR + CO_2_ while we measured brain activity in response to pneumatic skull taps (a validated method of vestibular stimulation). We also measured mobility and balance performance several times before and after the intervention. We found support for adaptive neural changes within the vestibular system during bed rest that subsequently recovered in several cortical and cerebellar regions. Further, there were multiple brain regions where greater pre- to post- *deactivation* was associated with *reduced* pre- to post- balance declines. That is, increased *deactivation* of certain brain regions associated with *better* balance post-HDBR + CO_2_. We also found that, compared to HDBR alone (*n* = 13 males; 29 ± 3 years) HDBR + CO_2_ is associated with greater increases in activation of multiple frontal, parietal, and temporal regions during vestibular stimulation. This suggests interactive or additive effects of bed rest and elevated CO_2_. Finally, we found stronger correlations between pre- to post- HDBR + CO_2_ brain changes and dependence on the visual system during balance for subjects who developed signs of Spaceflight-Associated Neuro-ocular Syndrome (SANS). Together, these findings have clear implications for understanding the neural mechanisms of bed rest and spaceflight-related changes in vestibular processing, as well as adaptation to altered sensory inputs.

## Introduction

Microgravity exposure poses unique challenges to human physiology: astronauts encounter body unloading, headward fluid shifts, altered vestibular and proprioceptive inputs, inflight and postflight spatial disorientation (Young et al., 1984), and confined quarters with carbon dioxide (CO_2_) levels up to more than ten times higher than those on Earth (Law et al., 2014). Upon return to Earth, astronauts present with multi-systemic consequences, such as declining bone (Sibonga, 2013) and muscle mass (LeBlanc et al., 1995; Stein, 2013), cardiovascular changes (Hargens and Richardson, 2009), and mobility and balance difficulties (Mulavara et al., 2010; Cohen et al., 2012; Wood et al., 2015). Here we focus on the neural vestibular consequences of a spaceflight analog environment, as well as the neural mechanisms underlying declines in vestibularly mediated mobility and balance.

Animal studies have demonstrated peripheral vestibular changes with spaceflight; for instance, utricular afferents become hypersensitive to translational accelerations after return to Earth (Boyle et al., 2001). Although the specific mechanisms for these changes remain unknown, one possibility is that the brain reinterprets afferent sensory input during flight due to the lack of a gravitational reference vector for the otoliths. After return to Earth, this re-interpretation is in conflict with Earth’s gravitational environment and results in postflight vestibular dysfunction (e.g., balance difficulties), followed by slow re-adaptation over the days and weeks following spaceflight (Young et al., 1984; Parker et al., 1985; Mulavara et al., 2010). Astronauts also present with decreased skin sensitivity on the soles of the feet following spaceflight (Lowrey et al., 2014), which has been attributed to in-flight sensory reweighting (i.e., the process of adjusting the magnitude of different sensory contributions to motor control) (Assländer and Peterka, 2014) in compensation for unreliable vestibular inputs in microgravity. A single-subject case study (Demertzi et al., 2016) and recent study of 11 cosmonauts (Pechenkova et al., 2019) examining resting-state and task-based functional magnetic resonance imaging (fMRI) connectivity found evidence for vestibular cortex reorganization and multisensory reweighting following long-duration spaceflight. This work provides preliminary evidence of flight-related central vestibular plasticity. Taken together, it is likely that spaceflight factors influence the neural correlates of vestibular processing; however, the precise mechanisms underlying such changes require further study.

Head-down-tilt bed rest (HDBR) is a common spaceflight analog intervention that permits ground-based study of how axial body unloading alters sensory inputs that subsequently impact neural vestibular processing and vestibular system plasticity. Subjects remain in bed with their head tilted down six degrees to mimic a subset of spaceflight consequences including headward fluid shifts, arterial pressure changes, axial body unloading, and reduced somatosensory input. Although gravitational vector input does not change during HDBR, there is evidence that, similar to spaceflight, axial body unloading contributes to sensory reweighting (Moore et al., 2010; Mulavara et al., 2018; Yuan et al., 2018b). Even though HDBR does not directly affect vestibular inputs, sensory reweighting is thought to affect neural vestibular processing during HDBR; more specifically, the vestibular nuclei receive inputs from the vestibular organs, in addition to proprioceptive signals from the limbs (Fredrickson et al., 1966; Rubin et al., 1979; Yates et al., 2000; Jian et al., 2002). If either vestibular or somatosensory inputs appear to be incorrect or abnormal, the central nervous system may use information from the other system to compensate and maintain performance (Bles et al., 1984; Dieringer, 1995; Horak and Hlavacka, 2001; Carriot et al., 2015). Thus, during HDBR, in the absence of normal somatosensory inputs to the foot, vestibular processing appears to be altered, with vestibular cues weighted more heavily (Mulavara et al., 2018). HDBR also results in reduced functional mobility and decreased postural stability, which are both behaviors that depend upon the vestibular system and multisensory integration (Reschke et al., 2009; Mulder et al., 2014; Koppelmans et al., 2015, 2017; Miller et al., 2018; Mulavara et al., 2018). Thus, taken together, HDBR provides an effective environment for studying neural vestibular adaptation and has applications for both space travel and for better understanding plasticity of the vestibular system and multisensory integration.

In recent years, several fMRI-compatible vestibular stimulation methods, including auditory tone bursts and pneumatic skull taps, have been used to map central vestibular processing networks (Schlindwein et al., 2008; Noohi et al., 2017). Two meta-analyses have revealed a diffuse vestibular processing network, including portions of insular cortex, premotor cortex, inferior parietal cortex, cingulate cortex, and the superior temporal gyri (Lopez et al., 2012a; Zu Eulenburg et al., 2012). However, the most commonly activated regions across several different vestibular stimulation methods were the parietal opercular area (“OP2”) and retroinsular cortex; consequently, these regions are sometimes referred to as “vestibular cortex,” and considered to be the core regions responsible for vestibular processing. In the present work, we stimulated the vestibular system during fMRI using pneumatic skull taps, which we have previously validated in young (Noohi et al., 2017) and older adults (Noohi et al., 2019), and successfully employed in our past HDBR work (Yuan et al., 2018b). Pneumatic skull taps elicit both activation in the vestibular cortex and deactivation in cross-modal sensory regions. Across both young and older adults, we have found associations between greater *deactivation* of certain subcortical and cerebellar regions in response to vestibular stimulation and *better* static balance performance (Noohi et al., 2019). This suggests the importance of both brain activation and deactivation, potentially reflecting sensory reweighting, for successful vestibular functioning.

In our past HDBR work, using this skull tap technique, we identified longitudinal brain changes suggestive of upregulation of vestibular processing in response to reduced somatosensory input during 70 days of HDBR (Yuan et al., 2018b). We also found associations between increased frontal, parietal, and occipital brain activity and greater HDBR-related mobility and balance declines, suggestive of reduced neural efficiency post-HDBR (Yuan et al., 2018b). Further, we identified post-HDBR increases in resting state connectivity for a network including the vestibular cortex and the cerebellum (Cassady et al., 2016). These findings indicate that the neural correlates of vestibular processing are altered with HDBR and have functional implications for vestibularly mediated behaviors.

No previous work has investigated the neural correlates of vestibular processing during an intervention combining HDBR with elevated CO_2_, which would better mimic the actual conditions on the International Space Station (ISS). Among other effects, exposure to heightened CO_2_ increases blood flow to the brain (at least initially) due to cerebral vasodilation (Atkinson et al., 1990; Zhou et al., 2008) and mildly impairs visuomotor function (Manzey and Lorenz, 1998). Although *reduced* blood levels of CO_2_ during voluntary hyperventilation have been associated with increased postural sway (Sakellari et al., 1997), it is unknown how *elevated* atmospheric CO_2_ interacts with central or peripheral vestibular processing.

Here we used fMRI to test changes in the neural response to vestibular stimulation with 30 days of HDBR paired with elevated CO_2_ (which we refer to as “HDBR + CO_2_”). We hypothesized that similar brain changes would emerge compared to our past work (referred to as “HDBR”), including evidence for HDBR-related upregulation of vestibular networks and reduced neural efficiency. Further, we anticipated that the interaction of HDBR and elevated CO_2_ would result in additive neural effects.

We addressed three primary aims in this small pilot sample (*n* = 11): (1) assess the time course of changes in the neural correlates of vestibular processing and recovery patterns with HDBR + CO_2_; (2) examine the functional consequences of HDBR + CO_2_ by associating brain changes with mobility and balance declines; and (3) characterize how HDBR + CO_2_ differentially affects the neural correlates of vestibular processing compared to HDBR alone. We developed an additional, exploratory aim (4) after about half of the HDBR + CO_2_ subjects developed signs of Spaceflight-Associated Neuro-ocular Syndrome (SANS) (Laurie et al., 2019), a condition which manifests with symptoms such as optic disk edema and is estimated to affect between approximately 16 and 50 percent of long-duration astronauts (i.e., those who have completed an ISS mission, which typically last for about 6 months) (Stenger et al., 2017). We characterized subgroup differences between those HDBR + CO_2_ subjects who did and did not develop signs of SANS.

## Materials and Methods

### HDBR + CO_2_

#### Participants

Eleven healthy individuals (six males, five females; mean ± SD age = 34 ± 8 years) provided their written informed consent and participated in 30 days of HDBR + CO_2_. This intervention was implemented within the larger study, VaPER (Visual impairment intracranial pressure and Psychological:envihab Research), in which separate investigators evaluated other physiological systems. All study procedures were approved by the local ethical commission of the regional medical association, Ärztekammer Nordrhein, as well as the University of Florida and NASA Institutional Review Boards.

#### Testing Timeline

**FIGURE 1:**
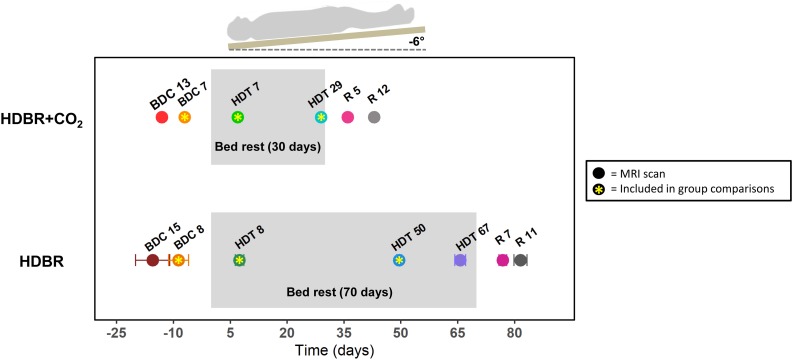
Testing timeline. **Top:** testing timeline for the HDBR + CO_2_ group, who completed 30 days of head-down-tilt bed rest (HDBR) with 0.5% elevated atmospheric CO_2_. **Bottom:** testing timeline for the HDBR group, who completed 70 days of HDBR with normal atmospheric CO_2_ levels. BDC, baseline data collection; HDT, head-down-tilt bed rest; R, recovery. Circles indicate the day for each MRI scan. Circles with asterisks represent the three time points used to create intercept and slope images for between-group comparisons. All HDBR + CO_2_ subjects completed MRI scans on exactly the same days with respect to bed rest. There was some variability in testing days for the HDBR group; average day is plotted, with error bars indicating standard deviation. Mobility and balance data were collected at all time points for the HDBR + CO_2_ group, with the exception of HDT 7 and HDT 29 (i.e., participants did not complete standing tasks *during* bed rest). One additional mobility and balance data collection took place on R0 for the HDBR + CO_2_ group within ∼3 h of first standing up. Mobility and balance scores at BDC 7 and R 0 were used for brain-behavior correlations with MRI data from BDC 7 and HDT 29.

Subjects were admitted to:envihab at the German Aerospace Center (Deutsches Zentrum für Luft- und Raumfahrt, DLR) in Cologne, Germany 14 days before the start of HDBR + CO_2_. During this time, they completed two baseline data collection (*BDC*) sessions (Figure 1). Subjects then underwent 30 days of six-degree HDBR with approximately 0.5% (partial pressure = 3.8 mmHg) elevated atmospheric CO_2_ (*HDT*), to match average ISS conditions (Law et al., 2014). Subjects kept a “strict” head-down-tilt position at all times, verified by 24/7 video monitoring. Subjects were instructed to always keep at least one shoulder in contact with the mattress. They were not permitted to use a pillow or to raise or stretch their legs aside from standardized physiotherapy sessions. Subjects remained at the facility for 14 days after HDBR + CO_2_ and completed two recovery (*R*) data collection sessions during this time.

fMRI images were collected at six time points: two times pre-, two times during, and two times post-HDBR + CO_2_ (Figure 1). Subjects completed mobility and balance testing on the same days as fMRI scans, except for the time points during HDBR + CO_2_. Subjects completed one additional mobility and balance testing session on the first recovery day (R0). One individual began testing late and thus did not complete BDC 13; however, this individual did complete the second baseline session (BDC 7).

### Head-Down-Tilt Bed Rest

#### Participants

Thirteen healthy individuals (all males; mean ± SD age = 29 ± 3 years) provided their written informed consent and participated in 70 days of HDBR. All study procedures were approved by the University of Michigan, University of Texas Medical Branch, and NASA Institutional Review Boards. These subjects represent a subset of the 18 total HDBR participants who received the same mode of vestibular stimulation caused by pneumatic skull taps as the HDBR + CO_2_ cohort. There were no significant age differences between the HDBR + CO_2_ and HDBR participants, and both cohorts passed a minimum physical fitness standard (i.e., an Air Force Class III equivalent physical examination) to participate (Lee et al., 2019a).

#### Testing Timeline

Participants were admitted to the NASA bed rest facility at the University of Texas Medical Branch, Galveston, TX, United States and completed two *BDC* sessions in the two weeks prior to starting HDBR (Figure 1). Participants underwent 70 days of HDBR with normal atmospheric CO_2_ (*HDT*). They maintained a six-degree head-down-tilt at all times except for 30 min during each meal, when they were allowed to support their head with their hand. Subjects remained at the facility for 14 days after HDBR and completed two recovery (*R*) data collection sessions during this time. All participants were a part of larger bed rest studies; thus, the timelines for HDBR + CO_2_ and HDBR were restricted by NASA and not identically matched between the two studies.

fMRI images were collected at seven time points: two times pre-, three times during, and two times post-HDBR (Figure 1). As we have previously reported neural vestibular changes with this intervention (Yuan et al., 2018b), here we use these fMRI data only for group comparisons with the HDBR + CO_2_ group. We examine only the fMRI scans from BDC 8, HDT 8, and HDT 50, as these points fell closest in time to those collected pre- and during bed rest for the HDBR + CO_2_ group and allowed us to compare slopes of change over time in the two groups. See Section “HDBR + CO_2_ vs. HDBR Group Comparisons” for details on this analysis approach for making between-group comparisons that account for these differing testing timelines.

### fMRI Data Collection

#### fMRI Acquisition

For the HDBR + CO_2_ group, fMRI scans were collected on a 3 Tesla Siemens MRI scanner. A gradient echo T2^∗^-weighted echo-planar imaging sequence was used to collect the fMRI scans: TR = 2.5 s, TE = 32 ms, flip angle = 90°, FOV = 192 × 192 mm, matrix = 64 × 64, slice thickness = 3.5 mm, voxel size = 3 × 3 × 3.5 mm^3^, 37 slices, 96 volumes. A T1-weighted gradient-echo pulse sequence was also collected with parameters: TR = 1.9 s, TE = 2.4 ms, flip angle = 9°, FOV = 250 × 250 mm, matrix = 512 × 512, slice thickness = 1.0 mm, voxel size = 0.49 × 0.49 × 1.0 mm^3^, 192 slices. Participants maintained the head-down-tilt position at all times using a foam wedge in the scanner. In addition, 0.5% CO_2_ was continuously supplied during the HDBR + CO_2_ intervention phase (through a mask and tank system when subjects were out of the environmentally controlled wing of the building).

For the HDBR group, fMRI scans were collected on a different 3 Tesla Siemens MRI scanner. A gradient echo T2^∗^-weighted echo-planar imaging sequence was used to collect the fMRI scans: TR = 3.66 s, TE = 39 ms, flip angle = 90°, FOV = 240 × 240 mm, matrix = 94 × 94, slice thickness = 4 mm, slice gap = 1 mm, voxel size = 2.55 × 2.55 × 5.0 mm^3^, 36 slices, 66 volumes. A T1-weighted gradient-echo pulse sequence was also collected with parameters: TR = 1.9 s, TE = 2.49 ms, flip angle = 9°, FOV = 270 × 270 mm, matrix = 288 × 288, slice thickness = 0.90 mm, voxel size = 0.94 × 0.94 × 0.90 mm^3^, 192 slices. The HDBR participants did not maintain the head-down-tilt position in the scanner; they were supine instead.

#### Vestibular Stimulation

For both the HDBR + CO_2_ and the HDBR cohorts, subjects received vestibular stimulation during fMRI. Subjects received skull taps via a pneumatic tactile pulse system [MR-compatible Pn Tactile Pulse System (PnTPS), Engineering Acoustics Inc.] placed over the lateral cheekbones (Noohi et al., 2017; Yuan et al., 2018b). The skull tapper used compressed air (50–55 psi) to power a small piston that delivered low-force taps (0.6 kg) to the cheekbone. We have recently shown that this approach is well tolerated by subjects, it activates vestibular cortical regions, it results in vestibular-evoked myogenic potentials in eye muscles, and it does not cause excessive head motion (Noohi et al., 2017).

Taps were delivered at 1 Hz, and each tapping block contained 24 taps. Both groups completed one fMRI run with five 24-s blocks of taps on the left cheekbone. Each block of taps was preceded and followed by 20-s rest periods. The HDBR group also completed a second run with taps to the right cheekbone; however, here we examine only the HDBR left tap run to enable direct comparison to the HDBR + CO_2_ group. Of note, although the vestibular stimulation parameters and total sequence duration were identical between groups, as the HDBR + CO_2_ fMRI sequence included a faster TR and more volumes (TR = 2.5 s; 96 volumes) than the HDBR sequence (TR = 3.66 s; 66 volumes), we acquired more data and thus had more statistical power for the HDBR + CO_2_ group. This represents a potential limitation of the present work and is discussed further in Section “Limitations.”

For both groups, the force of the taps was sufficiently low that they did not induce head motion that was greater than for other task runs. No subject moved more than 2.1 mm within any run, which is smaller than the size of one voxel.

### fMRI Preprocessing and Subject-Level Analyses

#### Preprocessing

Image preprocessing was completed using Statistical Parametric Mapping 12 (SPM12, version 7219) (Ashburner et al., 2016) with MatLab R2016a, version 9.0. We used a standard SPM preprocessing pipeline for fMRI (Ashburner et al., 2016). All functional images were corrected for slice timing then realigned and resliced to correct for head motion. As an additional quality check, we used the Artifact Detection Tool (ART)^1^ with motion threshold = 2.5 mm and global brain signal Z threshold = 9. There were no within-session movement outliers for either group. Only one individual in the HDBR + CO_2_ group had a global intensity outlier present in 4 of 96 volumes for one session; we used the subject-level covariate outputted by ART to minimize effects of these volumes on group-level analyses.

After resetting the origins of each T1 image to the anterior commissure, the T1 images were coregistered to the mean functional image with separation of [2, 1 mm]. The T1 images were segmented using the SPM12 Dartel algorithm with a sampling distance of 1 mm. The forward deformation fields from the T1 segmentation were used to normalize the functional images and the T1 to MNI space. We used 7th degree B-spline normalization for optimal performance (Ashburner et al., 2016). The warped images were spatially smoothed with an 8 mm full−width at half−maximum three−dimensional Gaussian kernel.

#### Subject-Level Whole Brain Statistical Analyses

At the subject level, we calculated brain activity for each participant on a voxel-by-voxel basis for left cheekbone vestibular stimulation versus rest. We set the first level masking threshold to -infinity and masked out non-brain areas using the “mask_ICV.nii” SPM intracranial volume mask. This allowed for inclusion of all voxels in the first level general linear model (GLM), as opposed to the default SPM masking threshold of 0.80, which includes in the GLM only those voxels with a mean value ≥ 80% of the global signal. We included ART-derived head motion parameters as nuisance variables in the subject-level analyses.

#### Cerebellar Processing

To improve normalization of the cerebellum and avoid over-stretching (Diedrichsen, 2006; Diedrichsen et al., 2009), we applied specialized processing using portions of both the CEREbellum Segmentation (CERES) (Romero et al., 2017) pipeline and the Spatially Unbiased Infratentorial and cerebellar Template (SUIT) (Diedrichsen, 2006; Diedrichsen et al., 2009) pipeline. We used CERES to segment the cerebellum from each person’s structural T1-weighted image. We then reset the origin of each individual’s cerebellum segmentation in native space to fall within the space of the segment. This allowed us to coregister each subject’s native space segmentation to the SUIT.nii template. We created binary gray matter, white matter, and full cerebellar masks from the CERES native space output and then used the suit_normalize_dartel function to obtain the Affine transformation matrix and flowfield needed to normalize these images into SUIT space.

We coregistered all of the slice timing-corrected, realigned/resliced (but *not* normalized) whole brain images to the T1-weighted whole brain image that was entered into the CERES pipeline and re-ran the subject-level statistical analyses described above on these *non-*normalized whole brain images. Then, using the Affine transformation and flowfield from normalizing the structural cerebellar segments to SUIT space, as well as each subject’s native space full cerebellar mask, we applied suit_reslice_dartel to the whole brain functional images to reslice all of the images into SUIT space. Given the small size of cerebellar structures, we applied a 2 mm smoothing kernel to the final functional cerebellar images and masked all second-level statistical results with a binary version of the SUIT.nii template, to avoid any spillover off the cerebellum due to the spatial smoothing. We performed all second-level statistical analyses described below twice: once for the whole brain (excluding the cerebellum) and a second time for only the cerebellum.

### fMRI Group-Level Statistics

#### Neural Response to Vestibular Stimulation

To demonstrate that our pneumatic tapper method was eliciting the expected vestibular system response, we first tested the main effect of vestibular stimulation averaged across all sessions for the HDBR + CO_2_ participants at peak-level *p* < 0.0005 (uncorrected), extent threshold = 10 voxels, controlling for age and sex differences.

#### Time Course of Neural Vestibular Response to HDBR + CO_2_

**FIGURE 2:**
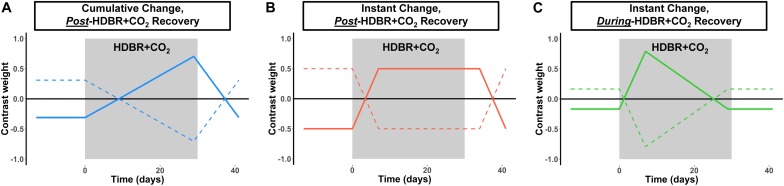
Hypothesized changes in neural vestibular response to HDBR + CO_2_. We hypothesized three different longitudinal patterns of brain change: **(A)** Cumulative change, in which brain changes would slowly increase over the course of HDBR + CO_2_, followed by recovery after the conclusion of HDBR + CO_2_. **(B)** Instant change, in which brain changes would immediately onset after the start of HDBR + CO_2_, followed by recovery post-HDBR + CO_2_. **(C)** Instant change, in which brain changes would immediately onset the start of HDBR + CO_2_, but begin recovery *during* bed rest. We tested each of these hypotheses by using the contrast vectors shown here as weights in our longitudinal statistical model which assessed brain changes over all six time points for the HDBR + CO_2_ group. Solid lines depict the positive version of each contrast; dotted lines depict the negative version of each contrast.

Similar to our past work (Yuan et al., 2016, 2018a,b), we tested for regions of immediate and cumulative change during bed rest followed by both quick and gradual recovery of brain activation patterns during vestibular stimulation across all six time points. We used flexible factorial analysis (SPM’s mixed model equivalent), controlling for age and sex, assuming independence between but not within subjects, and assuming equal variances between and within subjects (Gläscher and Gitelman, 2008; Kurth et al., 2010). We used several contrast vectors as weights for the statistical analyses to test the hypothesized relative level of activation during each session. Cumulative change (Figure 2A) was modeled as a progressive increase in activity across the course of HDBR + CO_2_, with a peak at the end of HDBR + CO_2_, and gradual restoration after the conclusion of HDBR + CO_2_. Immediate change (Figure 2B) was assumed to onset shortly after the start of HDBR + CO_2_, to maintain during HDBR + CO_2_, and to end shortly after the conclusion of HDBR + CO_2_. We hypothesized that recovery would be either quick (Figure 2C), occurring during bed rest (i.e., between HDT 7 and HDT 29), or that recovery would be more gradual, with altered brain activation patterns still evident at HDT 29 (Figures 2A,B). We tested for both increases and decreases in activation with each of these contrast shapes. To better detect within−subject changes with the complex longitudinal models used in this pilot study, the alpha level was set at *p* < 0.001 (uncorrected). We report clusters that are at least 10 voxels for the whole brain and *k* = 5 voxels for the cerebellum.

#### Correlations of Brain and Behavioral Changes With HDBR + CO_2_

We computed brain activation differences during vestibular stimulation between the final pre- HDBR + CO_2_ time point (BDC 7) and the final time point during HDBR + CO_2_ (HDT 29). We also computed the change in mobility and balance scores from BDC 7 to the first post-HDBR + CO_2_ time point, R0. To examine regions in which HDBR + CO_2_ brain changes were associated with changes in mobility and balance performance, we used a one-sample *t*-test model controlling for age and sex and included the behavioral change score as a covariate of interest. For each model, we used the Statistical Non-Parametric Mapping (SnPM version 13)^2^ (Nichols and Holmes, 2002) toolbox to run non-parametric permutation tests with 15,000 permutations, variance smoothing = 8 mm kernel for the whole brain analyses and 2 mm kernel for the cerebellar analyses, minimum cluster size = 10 voxels, and threshold = non-parametric *p* < 0.0005 (uncorrected). The SnPM toolbox is recommended for studies with small sample sizes that may not meet assumptions for parametric testing. The SnPM toolbox calculates pseudo *t*-statistic images and uses non-parametric permutation testing to assess for significance.

#### HDBR + CO_2_ vs. HDBR Group Comparisons

To examine differences in neural response to vestibular stimulation between bed rest with and without elevated CO_2_, we compared both baseline (i.e., intercept) differences between the HDBR + CO_2_ and HDBR groups, as well as the slope of change in brain activation across bed rest. As each cohort followed a different testing timeline, we compared the three time points that fell the closest together in time between the groups (indicated by asterisks in Figure 1). As in our previous work (Yuan et al., 2016, 2018b), we calculated a regression intercept and slope for each person using the scans from these three time points. The last image collected before the start of HDBR + CO_2_ or HDBR was treated as time = 0 days, assuming that pre-bed rest activation was stable. Calculating the regression intercept allowed us to examine baseline differences between groups, and calculating the regression slope allowed us to compare the rate of change in brain activation during vestibular stimulation between groups.

We used two sample *t*-tests to examine between-group differences in intercept and slope images. For all group comparisons, we used SnPM non-parametric permutation tests with 15,000 permutations, variance smoothing = 8 mm kernel for whole brain analyses and 2 mm kernel for cerebellar analyses, minimum cluster size = 10 voxels, and threshold = non-parametric *p* < 0.0005 (uncorrected). In each model, we accounted for age and sex differences. We excluded two individuals from the HDBR cohort from group analyses: one individual had severe artifacts in their HDT 50 scan, and another individual had abnormally high contrast values at the single-subject level, possibly also due to artifacts. Thus there were *n* = 11 subjects per group for group comparisons.

There were several differences between the HDBR + CO_2_ and HDBR images. Images were collected on different scanners, HDBR images showed evidence of greater orbitofrontal dropout compared to HDBR + CO_2_ images, and HDBR individuals presented with slightly smaller ventricles. To address this and remain conservative in our analyses, we do not report any between-group orbitofrontal results, and we report with caution one between-group result in close proximity to the ventricles. As these two groups represent highly unique cohorts who have undergone a rare, intensive bed rest intervention with nearly identical behavioral and neuroimaging protocols, we feel that it is still valuable to report on group differences between these two cohorts, although the results of these specific analyses should be interpreted with caution. As we previously reported on longitudinal neural vestibular changes and brain-behavior correlations for the HDBR group (Yuan et al., 2018b), the only HDBR results reported here are the group differences between HDBR + CO_2_ and HDBR.

#### SANS Versus No-SANS Group Comparisons

We performed two exploratory analyses to examine group differences between those HDBR + CO_2_ subjects who developed signs of SANS (SANS; *n* = 5; two males, three females) and those who did not (no-SANS; *n* = 6; four males, two females). First, we tested for differences between the intercept and slope images for each group. We conducted two-sample parametric *t*-tests with threshold *p* < 0.0005, *k* = 10. Non-parametric testing would not have been possible here, as less than 500 permutations exist for this combination of sample sizes.

Next, we tested for regions where the SANS versus no-SANS groups showed differences in the correlation between pre- to post-HDBR + CO_2_ brain change and pre- to post- change in the ratio between the balance—eyes open and balance—eyes closed condition. This ratio score was calculated as: (balance—eyes open score/balance—eyes closed score) ^∗^100 and provides a metric of the degree to which an individual relies on vision for maintaining quiet upright stance. Each of these balance tasks is described in Section “Balance Testing.” We selected to compare brain-behavior correlations only for this ratio score here because we previously identified significant differences between the two SANS subgroups on this measure, in which SANS individuals showed *greater increases* from pre- to post-HDBR + CO_2_ in their reliance on vision during balance compared to no-SANS individuals (Lee et al., 2019a). One of the five SANS subjects was excluded from this analysis due to outlier values for the balance—eyes closed condition (described in Section “Balance Testing”). Thus there were *n* = 4 for the SANS group and *n* = 6 for the no-SANS group.

### Mobility and Balance Testing: HDBR + CO_2_ Cohort Only

Although the HDBR + CO_2_ participants completed a battery of neurocognitive and sensorimotor assessments at each time point, here we focus on only mobility and balance testing, as these tasks were the most directly related to vestibular processing. We have previously published comprehensive behavioral profiles for both the HDBR + CO_2_ (Lee et al., 2019a) and HDBR groups (Koppelmans et al., 2015), as well as vestibular brain-behavior correlations for the HDBR group (Yuan et al., 2018b).

#### Functional Mobility Test (FMT)

The Functional Mobility Test (FMT) is sensitive to the effects of spaceflight (Mulavara et al., 2010) and to the effects of bed rest (Reschke et al., 2009; Koppelmans et al., 2017). The FMT requires subjects to arise from a seated position and walk through a 6-m × 4-m two-part obstacle course consisting of foam hurdles, pylons, and bars. The first part of the course was completed on a hard floor, and the second part was completed on medium-density foam. Participants were instructed to walk through the course as quickly as possible without touching any of the obstacles. Participants repeated the FMT 10 times per session on five different testing days (Figure 1). Here we analyze only the total time needed to complete the course for the first trial of each session, as we have found this measure to be the most sensitive to intervention-related change. We excluded one subject from FMT analyses, as the subject showed substantial pre- to post- slowing (> ± 2.5 standard deviations from the group average pre- to post- change) and exerted considerable influence on group-level statistics. Thus there were *n* = 10 subjects for analyses involving FMT.

#### Balance Testing

Participants completed three balance tasks: (1) balance—eyes open; (2) balance—eyes closed; and (3) balance—eyes closed dynamic head tilt. Details of these tasks have been previously described (Mulder et al., 2014). Participants stood on a foam pad on top of a force platform (Leonardo Mechanograph, Novotec Medical GmbH, Pforzheim, Germany). Participants were instructed to maintain a comfortable stance, keep their arms folded across their chest and remain in a stable, upright posture for 30 s. Foot markers on the foam pad were used to ensure consistent foot placement across trials and between subjects. For the first two conditions, participants kept their head erect and eyes either open or closed. For the eyes closed dynamic head tilt condition, participants kept their eyes shut and made head pitch motions of ±20°, synchronized to a 0.33 Hz metronome tone. Participants repeated all conditions three times during each testing session, and the order of conditions was semi-randomized to ensure that identical conditions did not repeat back-to-back. To minimize the effect of outlier trials, for each condition we examined the median score of the three trials. Scores are reported as equilibrium quotients (EQ), where 100% is a perfect score. EQ scores were calculated using instantaneous anterior-to-posterior peak-to-peak center-of-mass sway angle. For the eyes closed condition only, we excluded one subject, as the subject showed pre- to post-HDBR + CO_2_ declines > ± 2 standard deviations from the group average and exerted considerable influence on group-level statistics. Thus there were *n* = 10 subjects for analyses involving balance-eyes closed scores, but there were *n* = 11 subjects for all other balance analyses.

#### Statistical Analyses of Behavioral Data

For completeness, we tested pre- to post- HDBR + CO_2_ behavioral change, and we tested recovery for the mobility and balance tasks. In R 3.5.1 (R Core Team, 2013), using the last pre- bed rest time point (BDC 7) and the end of bed rest time point (HDT 29), we calculated a slope of pre- to post- performance change for each subject and conducted a one-sample *t*-test to determine if the group-average slope was different from 0. We also examined post-HDBR + CO_2_ recovery trajectories for the three post-bed rest time points using a linear mixed model with restricted maximum likelihood (REML) estimation via the “lme” function. The model included a random intercept for subject (to allow for different starting points for each person) and the fixed effect of time. In each case, we were interested in whether the fixed effect of time was significant; we tested a quadratic fit for time for each measure as well, but the model including the linear effect of time performed better in all cases.

## Results

### Neural Response to Vestibular Stimulation

**FIGURE 3:**
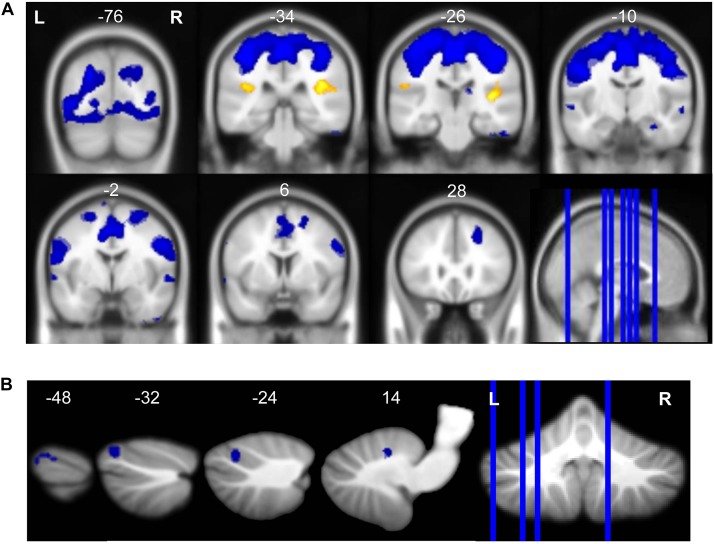
Neural response to vestibular stimulation. Vestibular stimulation resulted in activation of insular cortex and widespread deactivation including **(A)** frontal, temporal, occipital, subcortical, and **(B)** cerebellar regions. Whole brain and cerebellar results are overlaid onto MNI **(A)** and SUIT **(B)** standard templates, respectively; *p* < 0.0005, *k* = 10; red = regions of activation; blue = regions of deactivation.

Average BOLD signal during vestibular stimulation versus rest across all subjects and all time points is shown in Figure 3 to illustrate the neural response to the skull tap method. In line with previous work (Lopez et al., 2012b; Zu Eulenburg et al., 2012; Noohi et al., 2017; Yuan et al., 2018b) vestibular stimulation resulted in activation of clusters in the right and left insula (Table 1). Also, as anticipated, we observed widespread deactivation of frontal, temporal, occipital, subcortical, and cerebellar regions. These results demonstrate that our skull tap method was able to engage the vestibular system and produce the expected neural response.

**TABLE 1:** Regions of activation or deactivation in response to vestibular stimulation.

	**Extent (k)**	**Peak T-value**	**Peak *p*-value**	**MNI coordinates (mm)**		
				**x**	**y**	**z**
**Activation**						
*Insular*						
R Insula	374	5.269	1.092 × 10^–6^	38	−24	8
L Rolandic Operculum	150	5.032	2.580 × 10^–6^	−38	−36	20
**Deactivation**						
*Frontal*						
L Posterior-Medial Frontal Gyrus^a^	183,700	8.245	1.349 × 10^–11^	−6	−16	60
R Middle Frontal Gyrus	487	4.775	6.469 × 10^–6^	24	28	38
R Superior Medial Gyrus	12	3.791	1.821 × 10^–4^	2	44	36
*Temporal*						
R Inferior Temporal Gyrus	111	4.375	6.067 × 10^–6^	54	−30	−30
L Superior Temporal Gyrus	79	4.630	1.082 × 10^–5^	−62	−6	−2
R Superior Temporal Gyrus	133	4.500	1.699 × 10^–5^	60	−2	−4
R Parahippocampal Gyrus	37	4.224	4.372 × 10^–5^	28	−14	−24
R Inferior Temporal Gyrus	30	4.068	7.380 × 10^–5^	48	0	−48
R Olfactory Cortex	11	3.794	1.804 × 10^–4^	4	10	−12
*Occipital*						
L Superior Occipital Gyrus^a^	5,868	6.151	4.065 × 10^–8^	−22	−78	−32
*Subcortical*						
R Thalamus	25	4.163	5.373 × 10^–5^	14	−24	16
R Caudate Nucleus	11	3.889	1.328 × 10^–4^	8	14	8
*Anterior Cerebellum*						
R Cerebellar Lobule V	20	4.143	5.733 × 10^–5^	14	−54	−21
*Cerebellar Crus I*						
L Cerebellar Crus I	49	4.165	5.335 × 10^–5^	−24	−74	−29
L Cerebellar Crus I	10	4.033	8.289 × 10^–5^	−48	−72	−31

*Significance level set at *p* < 0.0005 and cluster size *k* = 10 for all analyses. Table shows all local maxima separated by more than 20 mm. Whole-brain results are listed first, followed by cerebellar results. Cortical regions were labeled using the AnatomyToolbox atlas via the SPM toolbox BSPMview. Cerebellar regions were labeled using the SUIT atlas. ^*a*^Portions of four deactivation clusters listed above passed Family Wise Error (FWE) < 0.05 correction:*

(1) L Posterior-Medial Frontal Gyrus: *k* = 5,036, FWE-corrected *p* = 2.203 × 10^–6^; MNI = −6, −16, 60

(2) L Superior Occipital Gyrus: *k* = 60, FWE-corrected *p* = 0.003; MNI = −22, −78, 32

(3) R Superior Frontal Gyrus: *k* = 32, FWE-corrected *p* = 0.006; MNI = 24, −10, 60

(4) L Inferior Occipital Gyrus: *k* = 24, FWE-corrected *p* = 0.014; MNI = −44, −78, 0

### Time Course of Neural Vestibular Response to HDBR + CO_2_

**FIGURE 4:**
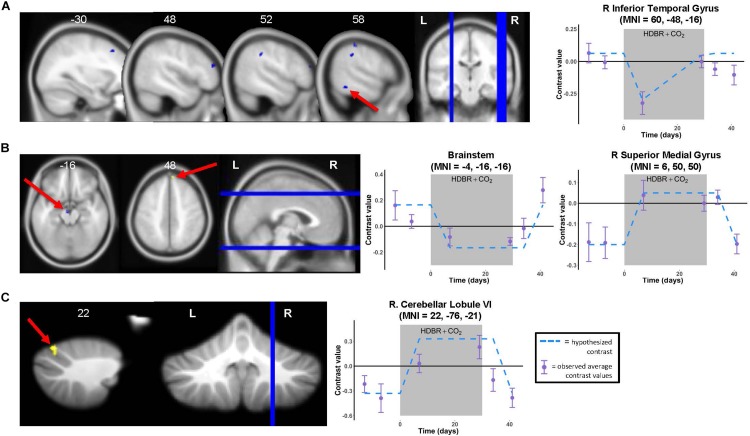
Time course of neural vestibular response to HDBR + CO_2_. Left panels: Regions showing whole brain **(A,B)** and cerebellar **(C)** longitudinal changes in activation during vestibular stimulation across the six time points. Whole brain and cerebellar results are overlaid onto MNI and SUIT standard templates, respectively; *p* < 0.001, *k* = 10 for whole brain analyses; *k* = 5 for cerebellar analyses. Three clusters survived *p* < 0.0005, *k* = 10 correction (see Table 2). Right panels: Example contrast values plotted for peak coordinate within the cluster with the largest *T* value in each case (cluster indicated by red arrow). Points represent group mean contrast values; error bars represent standard error. Dotted lines depict the hypothesized longitudinal contrasts for “instant decrease, during-HDBR + CO_2_ recovery” **(A)**, “instant decrease, post-HDBR + CO_2_ recovery” **(B)**, and “instant increase, post-HDBR + CO_2_ recovery” **(B,C)**.

We identified multiple longitudinal changes in the neural response to vestibular stimulation across HDBR + CO_2_, followed by recovery (Figure 4 and Table 2). Several frontal, parietal, and temporal regions (Figure 4A) showed immediate *decreases in activation* with HDBR + CO_2_, followed by recovery *during* the intervention and complete recovery by the final bed rest time point, HDT 29. For instance, right inferior temporal gyrus showed a conversion from *activation* to *deactivation* with the onset of HDBR + CO_2_, followed by recovery of *activation* of this region by HDT 29.

**TABLE 2:** Regions showing longitudinal increases and decreases in activation during vestibular stimulation across all six time points.

	**Extent (k)**	**Peak *T*-value**	**Peak *p*-value**	**MNI coordinates (mm)**		
				**x**	**y**	**z**
**Instant Decrease, During-HDBR + CO_2_ Recovery**						
*Frontal*						
R Middle Frontal Gyrus^a^	21	−3.953	1.238 × 10^–4^	48	46	20
L Middle Frontal Gyrus	15	−3.617	3.517 × 10^–4^	−30	26	44
*Parietal*						
R Supramarginal Gyrus	12	−3.999	1.071 × 10^–4^	58	−30	54
R Supramarginal Gyrus^a^	59	−3.835	1.798 × 10^–4^	52	−36	38
*Temporal*						
R Inferior Temporal Gyrus^a,b^	46	−4.271	4.463 × 10^–5^	60	−48	−16
**Instant Increase, Post-HDBR + CO_2_ Recovery**						
*Frontal*						
R Superior Medial Gyrus^b^	15	3.781	2.123 × 10^–4^	6	50	50
*Anterior Cerebellum*						
R Cerebellar Lobule VI^b^	9	4.093	7.938 × 10^–5^	22	−76	−21
**Instant Decrease, Post-HDBR + CO_2_ Recovery**						
*Subcortical*						
Brainstem^b^	15	−3.645	3.229 × 10^–4^	−4	−16	−16

*Clusters that emerged as significant were the same for the “instant, slow recovery” and “cumulative, slow recovery” increase and decrease contrasts. Thus here we report only the statistics for the instant increase and decrease contrasts. Significance level set at *p* < 0.001 and cluster size *k* = 10 for whole brain analyses and cluster size *k* = 5 for cerebellar analyses. Table includes all local maxima separated by more than 20 mm. Whole-brain results are listed first, followed by cerebellar results. Cortical regions were labeled using the AnatomyToolbox atlas via the SPM toolbox BSPMview. Cerebellar regions were labeled using the SUIT atlas. ^*a*^Portions of three “instant decrease, during HDBR + CO_2_ recovery” clusters survived *p* < 0.0005 and *k* = 10 thresholding:*

(1) R Inferior Temporal Gyrus: *k* = 24

(2) R Middle Frontal Gyrus: *k* = 12

(3) R Supramarginal Gyrus: *k* = 59

^*b*^Whole brain and cerebellar clusters with largest *T* value for each contrast; contrast values are plotted for the peak coordinate within each of these four clusters in Figure 4.

Several other regions showed patterns of fast change, with changes sustaining throughout HDBR + CO_2_ and not restoring until after the conclusion of bed rest. Right superior medial gyrus and right cerebellar lobule VI both showed *decreases in deactivation* and a conversion to *activation* with the start of bed rest, followed by recovery by 12 days post- bed rest. While neither of these clusters precisely overlaps with the regions that deactivated on average during vestibular stimulation (Figure 3 and Table 1), other nearby parts of the superior medial gyrus and right cerebellar lobule VI did significantly deactivate in response to vestibular stimulation.

One brainstem cluster showed a fast *decrease in activation* with bed rest, with a conversion to *deactivation* of this region during HDBR + CO_2_, followed by recovery. Of note, we did not find differences between the brain regions that emerged as significant for the “instant” versus “cumulative change post-HDBR + CO_2_ recovery” contrasts, so we have reported only results for the instant change post-HDBR + CO_2_ recovery contrasts.

### Functional Behavioral Implications

#### Mobility and Balance Changes With HDBR + CO_2_

**FIGURE 5:**
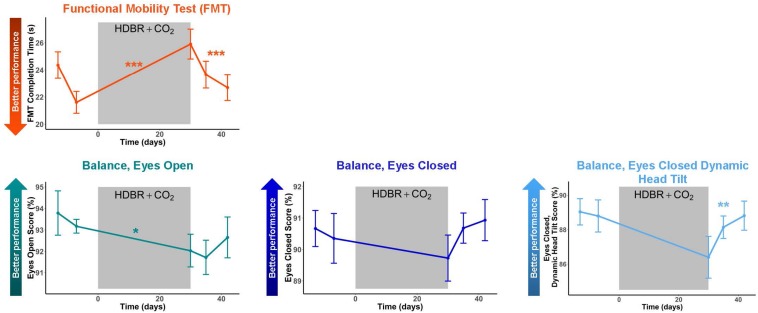
Mobility and Balance Changes with HDBR + CO_2_. Mobility and balance performance pre- and post-HDBR + CO_2_. In general, participants showed performance declines followed by recovery post-HDBR + CO_2_. The slope of behavioral decline was significant for FMT and a trend for the balance-eyes open condition. Participants showed a significant linear recovery trajectory for FMT and balance-eyes closed dynamic head tilt. Error bars represent standard error. ^∗^*p* < 0.10; ^∗∗^*p* < 0.05; ^∗∗∗^*p* < 0.001.

Subjects showed pre- to post-HDBR + CO_2_ declines in mobility, followed by a linear recovery pattern (Figure 5 and Table 3); that is, participants were slower to complete the FMT obstacle course post-HDBR + CO_2_, but sped back up by 12 days post-bed rest. The slope of decline in balance scores was only significant for the balance—eyes open condition; however, visually (Figure 5), there was a clear trend that HDBR + CO_2_ negatively impacted balance across all three tasks. Similarly, only the balance—eyes closed dynamic head tilt condition showed a significant linear recovery pattern post-HDBR + CO_2_, but again, visually, a recovery trend was evident post-bed rest for each of the balance tasks.

**TABLE 3:** Mobility and balance change with HDBR + CO_2_ and recovery.

	**Slope of Changes with HDBR + CO_2_**		**Fixed Effect of Time During HDBR + CO_2_ Recovery**		
	***t*(DF)**	***p***	**Recovery Day β**	**t(DF)**	***p***
Functional Mobility Test (FMT)	5.45(9)	**<0.001^∗∗∗^**	−0.260	−5.35(19)	**<0.001^∗∗∗^**
Balance—eyes open	−1.91(10)	**0.085^∗^**	0.057	0.86(21)	0.398
Balance—eyes closed	−0.51(9)	0.622	0.087	1.38(19)	0.182
Balance—eyes closed dynamic head tilt	−1.74(10)	0.113	0.195	2.31(21)	**0.032^∗∗^**

*^∗^*p* < 0.10 (trend); ^∗∗^*p* < 0.05; ^∗∗∗^*p* < 0.001.*

#### Brain—Behavior Correlations

**FIGURE 6:**
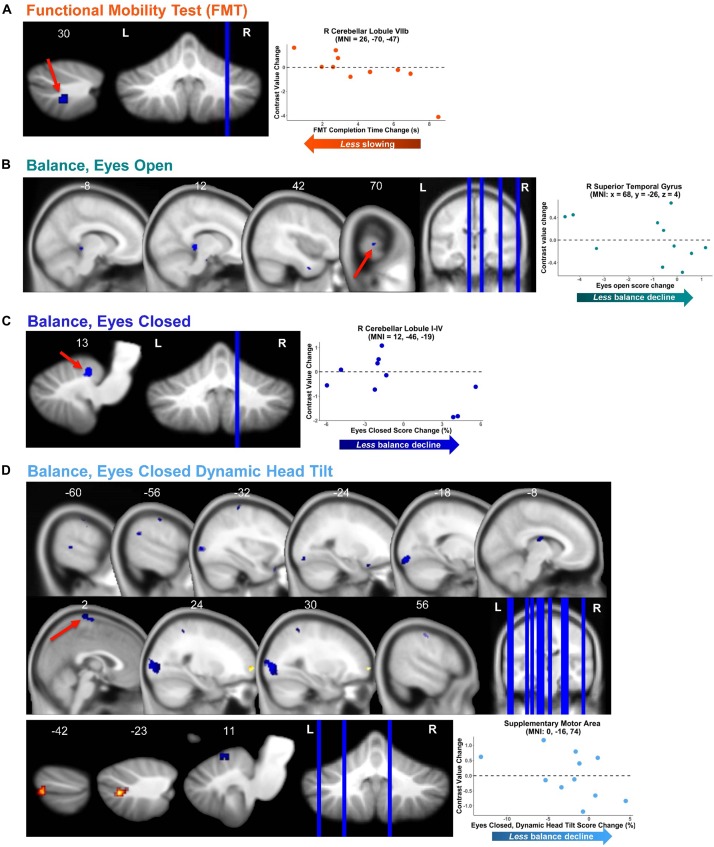
Functional behavioral consequences. Brain—behavior correlations for functional mobility test (FMT; **A**) and three balance tasks **(B–D)**. Brain change was calculated as the difference in brain activation from pre-HDBR + CO_2_ (time BDC 7) to the end of HDBR + CO_2_ (time HDT 29). Behavior change was calculated as the difference in mobility and balance performance from pre-HDBR + CO_2_ (time BDC 7) to post-HDBR + CO_2_ (time R0). Whole brain and cerebellar results are overlaid onto MNI and SUIT standard templates, respectively; non-parametric *p* < 0.0005, *k* = 10. Warm colors indicate regions of positive correlation between brain change and behavior change. Cool colors indicate regions of negative correlation between brain change and behavior change. Right side correlation plots include contrast values extracted from peak coordinate inside an example cluster (indicated with red arrows), graphed against behavior change score.

We identified several dozen regions for which pre- to post-HDBR + CO_2_ change in neural response to vestibular stimulation correlated with pre- to post- change in mobility and balance performance (Figures 6A–D and Table 4). In general, across all tasks and almost all clusters, we found that *greater deactivation* of various brain regions was associated with *reduced decline* or even improvement in behavioral measures. For instance, for the balance—eyes open condition, we found that *greater deactivation* of the right superior temporal gyrus (Figure 6B) associated with *less* balance decline and even balance improvement for a few individuals. That is, those with the greatest *decreases in activation* or *increases in deactivation* of this region had the *best* post-HDBR + CO_2_ balance performance. Similarly, for the eyes closed and eyes closed dynamic head tilt balance conditions, we found that *greater* pre- to post- *deactivation* of right cerebellar lobule I-IV and supplementary motor area, respectively, was associated with *less balance decline* or even balance improvement (Figures 6C,D).

**TABLE 4:** Regions showing associations between pre- to post-HDBR + CO_2_ differences in behavioral scores and pre- to post-HDBR + CO_2_ change in brain activation during vestibular stimulation.

	**Extent (*k*)**	**Peak *T*-value**	**Non-parametric Peak *p*-value**	**MNI coordinates (mm)**		
				**x**	**y**	**z**
**FMT^a^, negative association**						
*Posterior Cerebellum*						
R Cerebellar Lobule VIIb^c^	23	5.110	2.000 × 10^–4^	26	−70	−47
**Balance—eyes open, negative association**						
*Temporal*						
R Superior Temporal Gyrus^c^	10	3.662	1.333 × 10^–4^	68	−26	4
R Inferior Temporal Gyrus	12	3.205	4.000 × 10^–4^	42	2	−36
*Occipital*						
R Lingual Gyrus	29	5.128	1.333 × 10^–4^	12	−32	0
*Subcortical*						
Brainstem	10	3.812	2.667 × 10^–4^	−8	−36	−4
**Balance—eyes closed^b^, negative association**						
*Anterior Cerebellum*						
R Cerebellar Lobule I-IV^c^	12	6.895	2.000 × 10^–4^	12	−46	−19
**Balance—eyes closed, dynamic head tilt, positive association**						
*Frontal*						
R Frontal Superior Orbital Cortex	55	4.196	2.000 × 10^–4^	28	66	0
*Cerebellar Crus*						
L Cerebellar Crus II	10	4.821	2.667 × 10^–4^	−22	−74	−41
L Cerebellar Crus I	15	4.103	6.667 × 10^–5^	−40	−76	−41
**Balance—eyes closed, dynamic head tilt, negative association**						
*Frontal*						
R Precentral Gyrus	17	5.112	4.667 × 10^–4^	56	−14	48
Supplementary Motor Area^c,d^	90	4.769	1.333 × 10^–4^	0	−16	74
L Frontal Inferior Orbital Cortex	20	3.754	1.333 × 10^–4^	−22	18	−22
L Precentral Gyrus^d^	14	3.524	4.667 × 10^–4^	−30	−32	70
*Parietal*						
L Postcentral Gyrus	14	3.553	4.000 × 10^–4^	−58	−20	50
*Temporal*						
R Fusiform Gyrus	549	6.929	1.333 × 10^–4^	32	−86	2
L Superior Temporal Gyrus	45	5.165	2.000 × 10^–4^	−54	−50	26
L Middle Temporal Gyrus	37	4.715	4.667 × 10^–4^	−60	−40	4
*Occipital*						
L Lingual Gyrus	144	6.071	1.333 × 10^–4^	−18	−92	−14
L Middle Occipital Gyrus^d^	51	5.560	2.667 × 10^–4^	−34	−90	6
*Subcortical*						
L Thalamus	47	4.555	6.667 × 10^–4^	−8	−14	20
*Anterior Cerebellum*						
Right Cerebellar Lobule V	12	6.018	2.000 × 10^–4^	10	−62	−11

*Significance level set at non-parametric *p* < 0.0005 and cluster size *k* = 10 for all analyses. Table includes all local maxima separated by more than 20 mm. Whole-brain results are listed first, followed by cerebellar results. Cortical regions were labeled using the AnatomyToolbox atlas via the SPM toolbox BSPMview. Cerebellar regions were labeled using the SUIT atlas. ^*a*^One outlier subject was excluded from FMT analyses so *n* = 10; see section “Materials and Methods.” ^*b*^One outlier subject excluded from balance—eyes closed analyses so *n* = 10; see section “Materials and Methods.” ^*c*^Indicates clusters for which values from peak coordinate within cluster are plotted against behavior change score in Figure 6. ^*d*^Peak coordinate in cluster falls within region of brain *deactivation* in response to vestibular stimulation (Figure 3 and Table 1).*

This relationship between *greater deactivation* and *reduced* behavioral decline held across the majority of brain regions that showed significant brain—behavior correlations, including sensorimotor cortex (i.e., supplementary motor area and postcentral gyrus), temporal cortex, occipital cortex, brainstem, and cerebellum (particularly, anterior cerebellum and crus I and II). In several cases, increased deactivation was found in regions that typically *deactivate* during vestibular stimulation (Figure 3 and Table 1); for instance, this was the case for supplementary motor area, postcentral gyrus, and occipital gyrus (i.e., clusters marked with superscript “d” in Table 4).

There were only a few regions where *reduced* pre- to post- *deactivation* was more beneficial for post- HDBR + CO_2_ mobility and balance performance. For instance, in the case of right cerebellar lobule VIIb (Figure 6A), *decreased deactivation* from pre- to post- was associated with *less* FMT slowing (i.e., less decline). Similarly, for the balance—eyes closed dynamic head tilt condition, *less deactivation* from pre- to post- in left cerebellar crus II was associated with *less* balance decline.

### HDBR + CO_2_ vs. HDBR Group Comparisons

#### Baseline (Intercept) Differences

**FIGURE 7:**
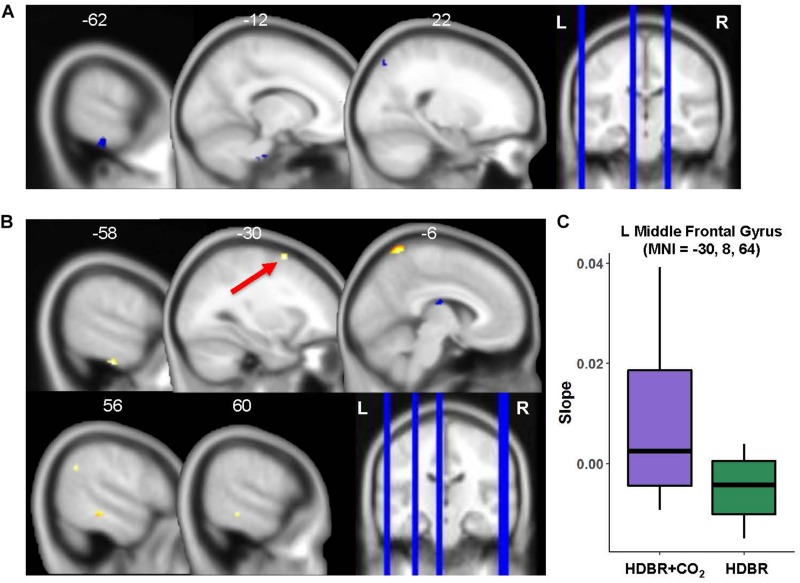
HDBR + CO_2_ vs. HDBR group comparisons. **(A)** Regions of intercept differences between HDBR + CO_2_ and HDBR groups. **(B)** Regions of slope differences between HDBR + CO_2_ and HDBR groups. Results overlaid onto MNI standard template; non-parametric *p* < 0.0005, *k* = 10. Cool colors indicate regions where the intercept or slope for the HDBR + CO_2_ group was numerically less than the intercept or slope for the HDBR group. Warm colors indicate regions where the slope for the HDBR + CO_2_ group was numerically greater than the slope for the HDBR group. **(C)** Example slope values extracted from L Middle Frontal Gyrus (i.e., the cluster with greatest *T* value for the HDBR + CO_2_ > HDBR contrast). Here, the HDBR + CO_2_ group showed *increased activation* of this region across bed rest, while the HDBR group showed *increased deactivation* of this region across bed rest.

Only three regions emerged where HDBR + CO_2_ and HDBR had baseline differences in neural response to vestibular stimulation: left inferior temporal gyrus, right superior occipital gyrus, and brainstem (Figure 7A and Table 5). That is, both groups produced similar neural responses to vestibular stimulation pre- bed rest, and thus between-group slope differences can likely be attributed to intervention effects.

**TABLE 5:** Regions with intercept differences between HDBR + CO_2_ and HDBR subjects.

	**Extent (*k*)**	**Peak *T*-value**	**Non-parametric Peak *p*-value**	**MNI coordinates (mm)**		
				***x***	***y***	***z***
**HDBR + CO_2_ < HDBR**						
*Temporal*						
L Inferior Temporal Gyrus	56	4.692	6.667 × 10^–5^	−62	−28	−28
*Occipital*						
R Superior Occipital Gyrus	10	3.798	2.668 × 10^–4^	22	−76	52
*Subcortical*						
Brainstem	67	3.752	3.333 × 10^–4^	−12	−22	−40

*Significance level set at non-parametric *p* < 0.0005 and cluster size *k* = 10. Table includes all local maxima separated by more than 20 mm. Cortical regions were labeled using the AnatomyToolbox atlas via the SPM toolbox BSPMview. There were no significant intercept differences for the cerebellum.*

#### Slope Differences

There were five clusters across frontal, parietal, and temporal cortex where the HDBR + CO_2_ group had a numerically *greater* slope of change in neural response to vestibular stimulation across the course of bed rest (Figure 7B and Table 6). Although no regions here overlapped with brain areas from the main effect analysis (Figure 3 and Table 1), these clusters were located in close proximity to regions that are expected to show *deactivation* during vestibular stimulation. In general, the HDBR + CO_2_ group showed *increases in activation* of these regions over the course of bed rest, as well as more within-group variability in neural response, compared to the HDBR group who generally showed *increases in deactivation* of these regions over the course of bed rest. For instance, in the left middle frontal gyrus (Figure 7C), the HDBR + CO_2_ subjects showed a switch from *deactivation* of this region during vestibular stimulation at BDC 7 to *activation* of this region at HDT 29. The HDBR group showed the opposite pattern, changing from *activation* to *deactivation* of this region.

**TABLE 6:** Regions with differences in slope of activation change with bed rest between HDBR + CO_2_ and HDBR subjects.

	**Extent (*k*)**	**Peak *T*-value**	**Non-parametric Peak *p*-value**	**MNI coordinates (mm)**		
				***x***	***y***	***z***
**HDBR + CO_2_ > HDBR**						
*Frontal*						
L Middle Frontal Gyrus^a^	20	4.373	1.333 × 10^–4^	−30	8	64
*Parietal*						
R Angular Gyrus	19	4.053	2.000 × 10^–4^	56	−54	34
L Precuneus	88	3.899	6.667 × 10^–5^	−6	−56	72
*Temporal*						
L Inferior Temporal Gyrus	27	4.313	6.667 × 10^–5^	−58	−22	−32
R Inferior Temporal Gyrus	28	4.120	2.667 × 10^–4^	60	−32	−12
**HDBR + CO_2_ < HDBR**						
*Subcortical*						
L Thalamus	29	4.073	6.667 × 10^–5^	−6	−14	20

*Significance level set at non-parametric *p* < 0.0005 and cluster size *k* = 10. Table includes all local maxima separated by more than 20 mm. Cortical regions were labeled using the AnatomyToolbox atlas via the SPM toolbox BSPMview. There were no significant slope differences for the cerebellum. ^*a*^Indicates cluster for which values from peak coordinate within cluster are plotted by group in Figure 7C.*

There was one cluster in the thalamus where the HDBR + CO_2_ group showed a *reduced* slope of change compared to the HDBR group. Here the HDBR + CO_2_ group exhibited *decreasing activation*, and ultimately *deactivation* of this region during vestibular stimulation at HDT 29, whereas the HDBR group exhibited a transition from *deactivation* of this region to *activation*.

### SANS vs. No-SANS Group Differences

Five of the 11 HDBR + CO_2_ participants developed signs of SANS, including optic disc edema. While this phenomenon is commonly reported following spaceflight (Lee et al., 2016), this is the first bed rest study to induce such effects (Laurie et al., 2019), possibly due to the careful testing for SANS symptoms and the strict head-down-tilt conditions, or the addition of elevated CO_2_. As SANS was not anticipated *a priori* but represents a substantial subgroup of the HDBR + CO_2_ cohort, we conducted two exploratory analyses of this unique sample.

**FIGURE 8:**
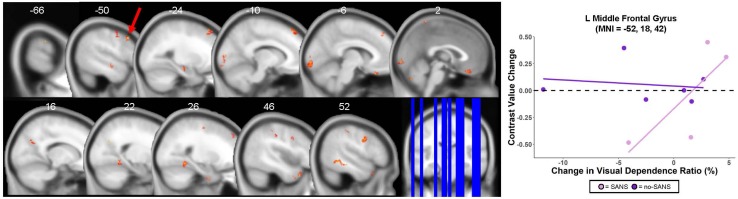
SANS vs. No-SANS Group Differences. **Left:** regions in which HDBR + CO_2_ participants who developed signs of Spaceflight-Associated Neuroccular Syndrome (SANS) showed a *stronger* correlation between pre- to post- change in brain activity during vestibular stimulation and pre- to post- change in balance ratio compared to those who did not develop signs of SANS (no-SANS). Balance ratio was calculated as: (balance—eyes open score/balance—eyes closed score) ^∗^ 100. Results overlaid onto MNI standard template; non-parametric *p* < 0.0005, *k* = 10. One of the five SANS subjects was excluded from analysis here due to outlier values for their eyes closed balance score; thus *n* = 4 for the SANS group and *n* = 6 for the no-SANS group. **Right:** correlation between brain changes and visual dependence balance ratio change for an example cluster; SANS subjects are shown in pink, no-SANS subjects are shown in purple. Regression lines are shown for each group for reference only and do not represent the results of any statistical tests.

At *p* < 0.0005 and *k* = 10, there were no regions of intercept or slope difference between the SANS and no-SANS participants. We previously found that SANS individuals increased their reliance on visual information during balance from pre- to post-HDBR + CO_2_ (Lee et al., 2019a); that is, SANS subjects had greater increases in their ratio of eyes open balance compared to eyes closed balance. Here we identified multiple frontal, parietal, temporal, and occipital regions where the SANS subjects showed stronger correlations with this balance ratio score compared to the no-SANS subjects (Figure 8 and Table 7). For instance, SANS subjects showed a stronger correlation between *greater activation* of left middle frontal gyrus (Figure 8) and *increased* pre- to post- balance ratio score. Several of these clusters included regions typically activated during vestibular stimulation (indicated with a superscript “d” in Table 7). There were no regions of stronger correlation for the no-SANS subjects.

**TABLE 7:** Regions where SANS subjects showed *greater* correlations compared to no-SANS subjects between pre- to post HDBR + CO_2_ change in visual dependence balance ratio and pre- to post-HDBR + CO_2_ change in brain activation during vestibular stimulation.

	**Extent (*k*)**	**Peak *T*-value**	**Peak *p*-value**	**MNI coordinates (mm)**		
				***x***	***y***	***z***
**SANS > no-SANS**						
*Frontal*						
L Middle Frontal Gyrus^a^	120	62.762	1.930 × 10^–7^	−52	18	42
L Superior Frontal Gyrus	121	47.969	5.650 × 10^–7^	−16	42	52
R Paracentral Lobule	11	35.986	1.780 × 10^–6^	2	−48	72
R Precentral Gyrus	56	26.726	5.826 × 10^–6^	48	6	38
L Rectal Gyrus	56	25.731	6.775 × 10^–6^	−2	30	-24
R Inferior Frontal Gyrus	11	23.941	9.027 × 10^–6^	56	20	2
R Precentral Gyrus^b^	22	18.232	2.661 × 10^–5^	28	−14	60
L Middle Orbital Gyrus	12	11.352	1.717 × 10^–4^	−38	40	4
R Superior Frontal Gyrus	17	11.211	1.802 × 10^–4^	28	42	44
L Precentral Gyrus	17	11.186	1.818 × 10^–4^	−52	−4	52
*Parietal*						
R Postcentral Gyrus^b^	35	38.070	1.422 × 10^–6^	38	−26	40
R Precuneus	34	33.849	2.272 × 10^–6^	16	−66	30
L Superior Parietal Lobule	20	29.913	3.719 × 10^–6^	−18	−62	46
R Postcentral Gyrus^b^	14	18.030	2.782 × 10^–5^	48	−28	48
L Postcentral Gyrus	27	17.676	3.009 × 10^–5^	−66	−20	34
R Postcentral Gyrus	12	14.543	6.500 × 10^–5^	66	−8	24
*Temporal*						
L Hippocampus	24	64.347	1.747 × 10^–7^	−28	−18	−18
R Medial Temporal Pole	32	27.635	5.099 × 10^–6^	46	20	−34
R Fusiform Gyrus	10	24.758	7.898 × 10^–6^	24	−4	−40
R Fusiform Gyrus	100	24.756	7.901 × 10^–6^	22	−48	−8
R Inferior Temporal Gyrus	86	21.709	1.332 × 10^–5^	52	−48	−6
R Inferior Temporal Gyrus	11	20.383	1.711 × 10^–5^	60	−54	−20
L Middle Temporal Gyrus	17	19.939	1.867 × 10^–5^	−50	−12	−10
L Fusiform Gyrus	11	19.920	1.874 × 10^–5^	−36	−46	−22
L Fusiform Gyrus	14	18.070	2.757 × 10^–5^	−38	−6	−38
R Medial Temporal Pole	13	16.837	3.647 × 10^–5^	46	10	−38
*Occipital*						
R Middle Occipital Gyrus^b^	20	27.454	5.235 × 10^–6^	30	−74	40
L Calcarine Gyrus	117	24.386	8.388 × 10^–6^	−6	−96	−6
L Mid-Occipital Gyrus^b^	10	12.832	1.063 × 10^–4^	−28	−84	14

*Significance level set at *p* < 0.0005 and cluster size *k* = 10. Table includes all local maxima separated by more than 20 mm. Cortical regions were labeled using the AnatomyToolbox atlas via the SPM toolbox BSPMview. There were no significant results for the cerebellum. ^*a*^Indicates cluster for which values from peak coordinate within cluster are plotted against behavior change score in Figure 8. ^*b*^Peak coordinate in cluster falls within region of brain *deactivation* in response to vestibular stimulation (Figure 3 and Table 1).*

## Discussion

### Key Findings

Here we identified changes in the neural correlates of vestibular processing with 30 days of HDBR + CO_2_. We found multiple regions where brain activation during vestibular stimulation changed quickly after participants started HDBR + CO_2_ and recovered either during or post-bed rest, providing support for adaptive plasticity of the vestibular system in response to altered sensory inputs. In multiple cases, *increased deactivation* of cortical and cerebellar areas was associated with *less* decline in balance from pre- to post-HDBR + CO_2_, suggesting that some of the adaptive neural changes during bed rest may benefit post-bed rest performance of vestibularly mediated behaviors. We found several differences for HDBR + CO_2_ compared to HDBR subjects, suggesting interactive or additive effects of bed rest and CO_2_. Finally, we noted differences in brain—behavior relationships for SANS versus no-SANS subjects, indicating the need for further study of bed rest-induced ocular symptoms.

### Time Course of Neural Vestibular Response to HDBR + CO_2_

Similar to our past work (Yuan et al., 2018b), we found multiple longitudinal changes in the neural response to vestibular stimulation, including in several areas in close proximity to regions typically involved in processing vestibular information, as well as in several regions that are not normally activated during vestibular stimulation. These responses could represent adaptive plasticity, in which the enhanced demands of neural processing of altered sensory inputs during HDBR + CO_2_ are requiring greater neural resources. For instance, the finding of *decreased deactivation* in right superior medial gyrus and right cerebellar lobule VI (which are anatomically near to regions that, on average, deactivated in response to vestibular stimulation) suggests a compensatory response. That is, functional brain regions that typically deactivate in response to vestibular input are deactivating *less* during exposure to an altered sensory environment, potentially to allow for additional brain pathways to aid in processing the novel sensory information. Here, more specifically, it could be that down-weighting of somatosensory input during HDBR, paired with upweighting of vestibular input due to vestibular-somatosensory convergence at the vestibular nuclei (Mulavara et al., 2012), is resulting in a higher neural processing demand. The fast recovery of several of these regions *during* bed rest suggests an ability of the vestibular system to adjust rapidly to such altered sensory conditions.

In contrast to our past work (Yuan et al., 2018b), we did not identify any unique regions of slow, cumulative brain changes; instead, we found clusters that were statistically significant for both the immediate and cumulative models. This suggests that interactive effects of CO_2_ with bed rest might accelerate neural vestibular changes. It could also be that CO_2_-related increases in cerebral perfusion enhanced the BOLD signal (Corfield et al., 2001) for HDBR + CO_2_ participants, making it easier to detect bed rest-related changes earlier during that intervention. Further, given that the HDBR + CO_2_ intervention was about half the duration of the HDBR intervention, it could be that some of the slow, cumulative brain changes that we previously identified require longer than 30 days to develop.

Previously we found that HDBR resulted in upregulation of the vestibular system (Yuan et al., 2018b), which we attributed to either increased sensitivity of the vestibular system during HDBR or to reduced neural efficiency, in which greater activation of vestibular cortical regions would be needed to process vestibular information during HDBR. Here, we did not find clear evidence for reduced neural efficiency (Yuan et al., 2018b). That is, we did not identify any HDBR + CO_2_-related *increases* in activation of vestibular cortical regions. This suggests that elevated CO_2_ may augment vestibular processing, as this environment did not produce the same longitudinal reduction in neural efficiency as HDBR alone.

### Functional Behavioral Implications

We identified predominantly regions for which *increased* pre- to post- *deactivation* during vestibular stimulation associated with *reduced* balance performance decline, or even performance improvement. This may represent adaptive plasticity during the HDBR + CO_2_ intervention, in which some individuals show an enhancement of the expected cortical deactivation response, paired with a dampening of activity in other brain regions that could interfere with processing of vestibular information. This adaptive change could then later manifest as superior post-bed rest balance due to underlying increased specificity of activation of vestibular cortex and deactivation of other sensory regions during the balance tasks. More specifically, it could be that the reduced plantar somatosensory input during HDBR + CO_2_ results in down-weighting of somatosensory input, but upweighting of vestibular input. While this reweighting is likely modulated at the level of the vestibular nuclei where the somatosensory and vestibular systems converge (Bles et al., 1984; Dieringer, 1995; Horak and Hlavacka, 2001; Carriot et al., 2015), this reorganization could plausibly manifest as increased deactivation of cortical sensorimotor processing regions, with those individuals who had the most successful reweighting processes presenting with enhanced preservation of balance abilities post- HDBR + CO_2_. Given these possible mechanisms, it thus makes sense that we found the most numerous brain-behavior correlations for the eyes closed dynamic head tilt condition, as this condition most specifically tasks the vestibular system.

We similarly found evidence of brain-behavioral relationships in our past HDBR work for vestibular processing (Yuan et al., 2018b) and for neural control of foot movement (Yuan et al., 2018a). Further, we previously identified several regions for which *better* balance (i.e., reduced postural sway while standing on one leg) correlated with *greater deactivation* of the brainstem, cerebellar lobule VI, and crus I and II across healthy young and older adults at one time point (Noohi et al., 2019). This fits with the present work, as here we found several brainstem and cerebellar regions for which *greater* post-bed rest *deactivation* associated with *better* balance. Together, these findings provide further support for the notion that those with a more refined neural response to vestibular stimulation in the scanner (e.g., including *greater deactivation* of brainstem and cerebellum) likely also produce a more refined neural response during balance tasks outside of the scanner and therefore perform better.

Similarly, several mobile neuroimaging studies have identified that older adults exhibit poorer balance paired with *increased* brain activation during vestibular stimulation (Karim et al., 2013; Lin et al., 2017). For instance, using functional near-infrared spectroscopy (fNIRS), Lin et al. (2017) found greater activation in frontal and occipital regions during vestibular stimulation for older compared to middle-aged adults. This suggests compensatory processes in which older adults require greater neural resources to process the same vestibular information. This fits with the present findings, as those who had the largest post-bed rest balance declines also showed bed rest-related *increases in activation* or *reductions in deactivation* across various cortical regions, including frontal and occipital regions. It could be that, similar to older adults, these individuals were recruiting extra brain regions to aid in processing vestibular information in the scanner and then engaging similar compensatory over-recruitment mechanisms outside the scanner during the post-bed rest balance assessments, which ultimately resulted in poorer balance performance.

### HDBR + CO_2_ vs. HDBR Group Comparisons

CO_2_-specific effects or interactive effects of bed rest and CO_2_ may be contributing to the identified differences in slope of activation change for the HDBR + CO_2_ group versus the HDBR group. CO_2_ is a strong vasodilator and, among other effects, results in increased blood flow to the brain (Atkinson et al., 1990; Zhou et al., 2008), as well as increased intensity of the blood oxygen level-dependent (BOLD) signal measured by fMRI (Corfield et al., 2001). This increased cerebral perfusion could be contributing to the identified group differences here. Elevated CO_2_ selectively favors frontal lobe perfusion (Bhogal et al., 2015); thus increased slope of change for frontal regions (e.g., middle frontal gyrus) among the HDBR + CO_2_ group could be particularly related to perfusion effects on the BOLD signal. As brain deactivation is also an active process, the widespread *increases in deactivation* that we noted (which were generally associated with better behavioral performance) could also be influenced by CO_2_-related perfusion effects.

It has been demonstrated that *decreased* CO_2_ (through voluntary hyperventilation) negatively impacts postural sway, resulting in unsteadiness of balance (Sakellari et al., 1997). Although the mechanisms here are unknown, it has been suggested that hyperventilation disrupts vestibular system compensation, including interfering with central and peripheral somatosensory signals from the lower limbs (Sakellari et al., 1997). Here the differential effects between HDBR + CO_2_ and HDBR subjects demonstrate that *increased* CO_2_ may also disrupt normal vestibular processing and vestibular compensatory mechanisms.

### SANS vs. No-SANS Group Differences

SANS subjects showed stronger correlations between pre- to post-HDBR + CO_2_ change in balance ratio score and brain changes. This suggests a relationship between SANS status and visual contributions to balance. Higher ratio scores are associated with more reliance on external visual cues for balance. As those with the greatest *activation* of regions such as middle frontal gyrus also showed the greatest pre- to post- *increase* in visual dependence, this could indicate reduced neural efficiency—in which these individuals are recruiting extra brain regions to aid in processing of the same vestibular information. This indicates reduced efficiency of the vestibular system that manifested behaviorally as greater reliance on the visual system during post-bed rest balance. These findings require validation in future studies to more clearly understand implications for the one third of astronauts who develop SANS (Lee et al., 2016).

### Limitations

Limitations of the present work include the small pilot sample, lack of ambulatory control group, and differences in scanning timeline and parameters between the two groups. As the HDBR + CO_2_ and HDBR groups were each part of separate, larger bed rest campaigns, the respective testing timelines were restricted by NASA and not matched between groups. Further, only males were included in the HDBR group, as these participants were control subjects for a different investigator’s testosterone supplementation study. Although the vestibular stimulation parameters and sequence duration were identical between the HDBR + CO_2_ and HDBR groups, these data were collected on two different Siemens scanners with two slightly different fMRI sequences. The HDBR + CO_2_ fMRI sequence included a faster TR and more volumes (TR = 2.5 s; 96 volumes) than the HDBR sequence (TR = 3.66 s; 66 volumes). This amounts to greater statistical power for the HDBR + CO_2_ group. While these differences represent a limitation of the between-group comparisons that we report here, multisite neuroimaging studies are increasing in popularity; such studies generally indicate that functional neuroimaging data is robust across sites (Costafreda et al., 2007; Biswal et al., 2010; Gountouna et al., 2010; Noble et al., 2017). Further, the analyses in the present work focus on differences in within-subject brain changes. That is, we have tested for between-group differences in within-person intercepts and slopes of change in brain activity. This makes the present results more robust to any introduced variance due to scanner or sequence differences.

Due to the limited pilot sample size, we use uncorrected *p*-values for the neuroimaging statistical tests to better detect within- and between-subject differences. Nonetheless, here we demonstrate for the first time the feasibility of characterizing vestibular brain changes during multiple weeks of bed rest combined with elevated CO_2_. We present compelling preliminary findings based on these highly unique data, which should be validated in future work.

It should also be noted that it is difficult to fully generalize these bed rest findings to spaceflight. HDBR + CO_2_ and HDBR mimic only some of the effects of spaceflight, such as body unloading, altered sensory input, and fluid shifts, but these analogs do not include all features of spaceflight that could impact vestibular processing. Finally, as is typical with the pneumatic skull tap method, the stimulus did not induce any vestibular perception, motion sensation, or movement in head position. Instead of using subjective perception of the vestibular stimulus, assessment of vestibular-evoked myogenic potentials in the eye muscles (oVEMPs) outside of the scanner was used to validate successful stimulation of vestibular organs.

### Applications to Spaceflight and Future Directions

The present findings document changes in functional vestibular processing with spaceflight analog environments. These findings support that spaceflight factors do likely influence the neural correlates of vestibular processing; however, there is limited past work investigating vestibular processing in astronauts. Two previous studies suggest changes from pre- to post- spaceflight in resting-state (Demertzi et al., 2016) and task-based connectivity (Pechenkova et al., 2019) in brain networks that support vestibular function. Our past work has identified disrupted white matter structural connectivity in several tracts that underlie sensory integration and vestibular processes and associations of these brain changes with balance declines (Lee et al., 2019b). For instance, we found that astronauts with the *largest* spaceflight-associated balance disruptions also had the *greatest* white matter declines in the superior longitudinal fasciculus (Lee et al., 2019b), which connects the temporoparietal and prefrontal cortices and is thought to subserve vestibular functions (Spena et al., 2006). However, no studies to date have tested spaceflight-related changes in functional brain activity during processing of vestibular information. To address this critical literature gap, an ongoing prospective study by our group is measuring brain activity with the pneumatic skull tap paradigm at two time points before and four time points after astronauts complete ISS missions. Comparing these results with the present work will help to elucidate how additional microgravity factors not induced by bed rest (e.g., an altered gravitational vector) might affect the neural correlates of vestibular processing.

## Conclusion

Here we demonstrate the feasibility of assessing longitudinal neural vestibular changes following 30 days of HDBR + CO_2_. We identify support for specific effects of combined HDBR + CO_2_ on vestibular processing, adaptive plasticity of the vestibular system during HDBR + CO_2_ followed by fast and slow recovery, and relationships between adaptive plasticity and spared behavioral performance post-HDBR + CO_2_. We note some differences between neural processing of vestibular information for HDBR + CO_2_ versus HDBR subjects, as well as implications for dependence on visual cues during balance for SANs versus no-SANS subjects. Together, these findings contribute to understanding of how the vestibular system adapts to altered sensory inputs and to understanding of how spaceflight may influence the neural correlates of vestibular processing.

## Data Availability Statement

The raw data supporting the conclusions of this article will be made available by the authors, without undue reservation, to any qualified researcher.

## Ethics Statement

The studies involving human participants were reviewed and approved by the local ethical commission of the regional medical association, Ärztekammer Nordrhein, as well as the University of Florida and NASA Institutional Review Boards. The patients/participants provided their written informed consent to participate in this study.

## Author Contributions

KH analyzed the vestibular fMRI data and mobility/balance behavioral data, created the figures and tables, and wrote the manuscript. JL collected and managed the data and participated in manuscript preparation. NG collected and analyzed the data. IK participated in project design and software development. YD collected and analyzed the data. JB, AM, and RS designed the project, secured funding, and led the interpretation and discussion of the results. All authors participated in revision of the manuscript.

## Conflict of Interest

The authors declare that the research was conducted in the absence of any commercial or financial relationships that could be construed as a potential conflict of interest. The reviewer RG declared a past co-authorship with several of the authors AM and JB to the handling Editor.

## Footnotes

1 www.nitrc.org/projects/artifact_detect/

2 http://warwick.ac.uk/snpm
